# *Eurosurveillance* reviewers in 2022

**DOI:** 10.2807/1560-7917.ES.2023.28.1.2301051

**Published:** 2023-01-05

**Authors:** Eurosurveillance editorial team

**Affiliations:** 1 European Centre for Disease Prevention and Control (ECDC), Stockholm, Sweden

**Keywords:** peer-review

The editorial team is grateful to all the experts who dedicated their time to peer-review our articles over the past 12 months. We wish to thank them and all those whose names are not listed below but gave helpful advice and support. We apologise to any reviewers whose names we may have missed.

Our reviewers in 2022 were:

Amos Adler; Rao Agam; Leonidas Alexakis; Sheikh Taslim Ali; Vanessa Allen; Erik Alm; Maria Joao Alves; Filipa Alves da Costa; Fátima Amaro; Maria an der Heiden; Aura Andreasen; Emmanuel André; Andrea Antinori; Alberto Antonelli; Brett Archer; Tommi Asikainen; Tatjana Avsic-Zupanc; Benjamin R Baer; Henry C Baggett; Tamas Bakonyi; Eszter Balla; Susan Ballard; Mathieu Bangert; Cyril Barbezange; Ian Barr; Ulrike Baum; Edward Belongia; Alexandre Belot; Anna Beltrame; Shmuel Benenson; Beatrice Bercot; Matthijs Berends; Laura Berneking; Debra Bessen; Pierre Bessiere; Julie Bettinger; Simona Bignami; Jonas Björk; Carina Blackmore; Lucille Blumberg; Benjamin Blümel; Máirín Boland; Idesbald Boone; Maria Laura Boschiroli; Herve Bourhy; Amanda Bradley-Stewart; Michael Brandl; Carina Brehony; Nathan J Brendish; Anne Brisabois; Sylvain Brisse; Basil Britto Xavier; Eeva Broberg; Kim Brolin; Caroline Bröjer; Laura Bubba; Alicia Budd; Michael Busch; Karina Butler; Leon Caly; Christine Campese; Rafael Cantón; Heather Carleton; Yehuda Carmeli; Jean-sebastien Casalegno; Kelsie Cassell; Alessandro Cassini; Nadim Cassir; Francesco Castelli; Jesus Castilla; Vincent Cattoir; Dominique Caugant; Orlando Cenciarelli; Muge Cevik; David Champredon; André Charlett; Remi Charrel; Cindy Chiu De Vazquez; Alexandra Choi; Michal Chowers; Nadia Ciampa; Jose Miguel Cisneros; Hazel Clothier; Bruno Coignard; Vittoria Colizza; Mélanie Colomb-Cotinat; John Conly; Jeff Connell; Martin Cormican; Ana Paula Coutinho Rehse; Benjamin Cowie; Benjamin Cowling; Fortunato D'Ancona; Gavin Dabrera; Viktor Dahl; Ghassan Dbaibo; Brechje de Gier; Marit de Lange; Franck de Laval; Elisabeth Delarocque-Astagneau; Alessandra della Torre; Oliver Denis; Charlotte Deogan; Brecht Devleesschauwer; Nolwenn Dheilly; Sofie Dhollander; Santina Di Bella; Antonino Di Caro; Liselotte Diaz Högberg; Frederika Dijkstra; Jac Dinnes; Gerhard Dobler; Margaret Doll; Lisa Domegan; Laurent Dortet; Jan Drexler; Christian Drosten; Julian Druce; Timothee Dub; Erwin Duizer; Jake Dunning; David Durrheim; Florence Débarre; Tim Eckmanns; Michael Edelstein; Androulla Efstratiou; Ehud Elnekave; Devy Emperador; Giulia Errico; Allahna Esber; Laura Espenhain; W. Douglas Evans; Daniel Faensen; James Fielding; Helen Fifer; Julie Figoni; Antonietta Filia; Jose Ramon Fiore; Robert Fischer; Brendan Flannery; ‪Daniele Focosi; Arnaud Fontanet; Laure Fonteneau; Nicoletta Formenti; Ron Fouchier; Nelly Fournet; Christina Frank; Sebastian Funk; Emmanouil Galanakis; Andrea Gervelmaeyer; Sarah Gerver; Isaac Ghinai; Yvonne Gilleece; Gary Ginsberg; Aharona Glatman-Freedman; Daniel Golparian; Maria Gossens; Céline Gossner; Magnus Gottfredsson; Rok Grah; M. Lindsay Grayson; Margrethe Greve-Isdahl; Martin P Grobusch; Thomas Gronthal; Thomas Grönthal; barbara Grützmacher; Claire Guinat; Bernardo Rafael Guzman Herrador; Stephan Göttig; Sebastian Günther; Stefan Hagel; Alvin X Han; Sonja Hansen; Lisa Hansen; Kayla Hanson; Thomas Harder; Aaron Harris; David M. Hartley; Henrik Hasman; Margaret Haworth-Brockman; Florian Heger; Janneke Heijne; Ulrich Heininger; Franz Heinz; Victoria Hernando; Marta Hernández-García; Megan Hofmeister; Mark Holmes; Martin Holt; Camilla Holten; Ralph Huits; Ralph Huits; Anders Husby; Anders Hviid; Michael Höhle; Judith Hübschen; Derval Igoe; Victoria Indenbaum; John PA Ioannidis; Gregorio Iraola; Takahiro Itaya; Jesus Iñigo; Stéphanie Jacquinet; Agatha Jassem; Claire Jenkins; Silvia Jimenez-Jorge; Kari Johansen; Annette Jurke; Annette Jurke; Stefanie Kadykalo; Günter Kampf; Tommi Karki; Taishi Kayano; Mária Kazimírová; Lee Kennedy-Shaffer; Gilbert Kersh; John Kinsman; Nishant Kishore; Esther Kissling; Masaaki Kitajima; Wioleta Kitowska; Lene Jung Kjær; Lene Jung Kjær; Sofieke Klamer; Irena Klavs; Petra Klepac; Anders Koch; Anke Kohlenberg; Britta Kohlmorgen; Archana Koirala; Shihoko Komine-Aizawa; Rolf Kramer; Marcela Krutova; Kevin Kuchinski; Anna Kuehne; EJ Kuijper; Michael Kundi; Sohvi Kääriäinen; Martin Kåberg; Csaba Ködmön; Hannah König; Raskit Lachmann; Nina Lagerqvist; Jeffrey Lazarus; Stéphane Le Vu; Nelson Lee; Jean-Daniel Lelièvre; Vivian Leung; Kathy Leung; Troels Lillebaek; Bruno Lina; Anders Lindblom; Adrian Lison; Florence Lot; Claudia Lucarelli; Åke Lundkvist; Yaniv Lustig; Irja Lutsar; Theodore Lytras; Outi Lyytikäinen; Joaquín López-Contreras Gonzalez; Emma Löf; Renke Lühken; Gonçalo Macedo; Raina MacIntyre; Guerrino Macori; Alexandra Mailles; John Mair-Jenkins; Anna Maisa; Gannon C.K. Mak; Jean-Michel Mansuy; Ulrich Marcus; Robby Markwart; Robby Markwart; Robby Markwart; Alexandra Marmor; Louise Marron; Katherine E. Marshall; Stacey W Martin; Iván Martínez-Baz; Sébastien Matamoros; Wesley Mattheus; Sebastian Maurer-Stroh; Andrea McCollum; Scott McDonald; James McMahon; Jim McMenamin; Adam Meijer; Anika Meinen; Roberto Melano; Angeliki Melidou; Tanya Melillo Fenech; Stefano Merler; Kevin Messacar; Eline Meyers; Chris Ka Pun Mok; Jose Molina-Mora; Monica Monaco; Orna Mor; Jacob Moran-Gilad; Alain Moren; Leah Moriarty; Joël Mossong; Aikaterini Mougkou; Laurent Moulin; Antons Mozalevskis; Judith Mueller; Maximilian Muenchhoff; Michio Murakami; Benjamin Murrell; David Muscatello; Otilia Mårdh; Kåre Mølbak; Tony Nelson; Sema Nickbakhsh; Andreas Nitsche; Harold Noel; Paulo Nogueira; Baltazar Nunes; Teresa Nygren; Sheila F. O'Brien; Joan O'Donnell; Sean O'Leary; Doris Oberle; Nicholas Ogden; Ajibola Omokanye; Joshua Osowicki; Juliette Paireau; Scott Pallett; Annalisa Pantosti; Anna Papa; John Papp; Dimitrios Paraskevis; Prabasaj Paul; Jean-Michel Pawlotsky; Lara Payne; Richard Pebody; Malik Peiris; Pasi Penttinen; Mariana Perez Duque; Carla Perrotta; Eskild Petersen; Patrizio Pezzotti; Germán Peñalva; Martin Pfeffer; Anastasia Pharris; Anastasia Phillips; Nick Phin; Roan Pijnacker; Rosa Pintó; Denis Piérard; Diamantis Plachouras; Mario Plebani; Catherine Plüss-Suard; Laurent Poirel; Piero Poletti; Stefano Pongolini; Leo Poon; Stephanie Popping; Bastian Prasse; Elisabeth Presterl; Henrieke Prins; Katarina Prosenc; Mirja Puolakkainen; Tina Purnat; Mario Ramirez; Ruwan Ratnayake; Giovanni Ravasi; Cameron Razieh; Gili Regev-Yochay; Hariharan Regunath; Ralf Reintjes; Jordi Rello; Ute Rexroth; Giovanni Rezza; Flavia Riccardo; Maria Luisa Ricci; Matteo Riccò; Wendy Rice; Maximilian Riess; Thomas V. Riley; Caterina Rizzo; Ilia Rochlin; Nash Rochman; Ana Rodrigues; Ana Rodrigues; Jesús Rodríguez-Baño; Maria Romay-Barja; Dan Romer; Angela Rose; John Rossen; Gian Maria Rossolini; Maja Rupnik; Jan Rupp; Kati Räisänen; Raquel Sabino; Örjan Samuelsen; Frank Sandmann; Jussi Sane; André Scherag; Gabrielle Schittecatte; Axel Schmidt; Madlen Schranz; Luigi Sedda; Philippe Selhorst; Luca Settanni; Azam Ali Sher; George Shirreff; Rami Sommerstein; Gianfranco Spiteri; Jean-Paul Stahl; John Stelling; Ellen Stobberingh; Franc Strle; Lorenzo Subissi; Jonathan Suk; Sheena Sullivan; Gang Sun; János Sándor; Emi Takashita; Heather Tate; Lara Tavoschi; Kjetil Elias Telle; Bernd-Alois Tenhagen; Karen Terio; Heidi Theeten; Craig Thompson; Valtyr Thors; Laura Tomassone; Sara Tomczyk; Ana Torres; Maria Elena Tosti; Alenka Trop Skaza; Sarah Tschudin-Sutter; Gro Tunheim; Yuki Uehara; Soren Uldum; Veronika Učakar; Nirma Vadlamudi; Chris Van Beneden; Wim Van Bortel; Michiel van Boven; Wim van der Hoek; Mark van der Linden; Marianne van der Sande; Sebastiaan van Hal; Jasper Van Heuverswyn; Angela van Hoek; Esther Van Kleef; Maaike van Mourik; Sonja van Roeden; Carmen Varela Martinez; Aisling Vaughan; Tomas Vega-Alonso; Pierre Verger; Sophie von Dobschuetz; Rengina Vorou; Jacco Wallinga; Jan Walter; Stella Watson; Guido Werner; David Whiley; Robert Whittaker; Micael Widerström; Andreas Widmer; Annelies Wilder-Smith; Hendrik Wilking; Thomas Williams; Christopher Williams; Carl-Heinz Wirsing von König; Sook San Wong; Jessica YT Wong; Ian Woolley; Tom Woudenberg; Cameron Wright; Stanley Xu; Barbara Yawn; Dana Yelin; Karene Yeung; Philipp Zanger; Peter Zarb; Petr Zeman; Dominik Zenner; Ye Zhang; Shi Zhao; Ruth Zimmermann; Kate Zinszer; Ljiljana Zmak; Julio Álvarez Sánchez.‬

